# Effects of specific physiotherapeutic exercises in a patient with a severe neuromuscular scoliosis with rigid spine syndrome: a case report

**DOI:** 10.1186/1748-7161-9-S1-P5

**Published:** 2014-12-04

**Authors:** Pamela Espinoza, Ignacio Dockendorff

**Affiliations:** 1 MEDS Sport Clinic, Santiago, Chile; 2 Clínica Alemana, Santiago, Chile

## Background

Non-idiopathic Scoliosis account for approximately 20% of scoliosis population and some cases need surgery. Exercises are important for the rehabilitation following fusion.

## Design

Case report.

## Aim

To show the improvements in the positive sagittal balance and flat back condition in a patient with a severe neuromuscular scoliosis (NMS) with Rigid Spine Syndrome (RSS) treated with specific physiotherapeutic exercises.

## Methods

A 16-year-old male patient diagnosed with severe NMS with RSS underwent to a posterior spinal fusion from T5 to L1 at 10 years-old. Surgery reduced the Cobb’s angle at the main thoracic, lumbar and the cervical curvature to half. Nonetheless, post-surgery complications appeared: the cervical curvature increased, positive sagittal balance appeared, hyperextension of the neck and head, flat back and kyphosis posture was developed. On October 2013 the patient started a program with specific exercises for scoliosis 2-3 times a week, based on rotational breathing principles from the Schroth method, Pilate’s exercises and physical therapy lumbopelvic stabilization exercises following the standard features of scoliosis rehabilitation schools.

## Results

Radiographies and documented pictures were taken at the beginning and at the sixth week of treatment. A comparison of these images shows an improvement in the flat back condition where the lumbar lordosis angle increased and positive sagittal balance was normalized. Currently, the patient is able to maintain this new posture by his own, however, he usually tends to fall into the misalignments postural vicious.

Conclusions

Short term results were observed in a difficult case of scoliosis using a convergent approach of different treatment techniques. Although Cobb’s angle remains, changes are evident at lateral radiography and documented pictures. Specific exercises could be an effective way to maintain or reduce the progression of the positive sagittal balance, improve the flat back condition and quality of life.
